# A Rare Case of Preiser Disease Affecting a Young Female

**DOI:** 10.7759/cureus.32955

**Published:** 2022-12-26

**Authors:** Rahul Salunkhe, Rushikesh Abhyankar, Ashwin Deshmukh, Ishan Shevate, Vellanki Sravan

**Affiliations:** 1 Orthopaedics and Trauma, Dr. D. Y. Patil Medical College, Hospital and Research Centre, Pune, IND

**Keywords:** preiser disease, proximal row, carpectomy, idiopathic, young female

## Abstract

Avascular necrosis of the carpal scaphoid is known as Preiser disease. Avascular necrosis is usually caused by non-traumatic or idiopathic etiology.

In this case report, we present the case of a 23-year-old female patient who came to our outpatient department with complaints of pain and swelling over the left wrist joint for seven months. The patient did not give any history of trauma or long-term steroid intake. Clinically, the patient had tenderness over the left anatomical snuff box. A plain radiograph of the wrist joint did not suggest any abnormality. MRI was done to confirm the diagnosis. MRI showed altered marrow signals in the scaphoid, which was suggestive of avascular necrosis of the scaphoid, also known as Preiser disease.

Proximal row carpectomy was done for the patient, and wrist range of motion exercises were started after one week postoperatively. Full range of motion of the wrist joint was achieved at three weeks postoperatively without no residual deformity.

## Introduction

As described by Preiser in 1910, Preiser disease is a rarefying osteitis of the scaphoid that he distinguished from scaphoid fracture [1]. He compared the condition with Kienbock’s disease and thought that the causes were similar. Collagen vascular disease, repetitive trauma, steroid therapy, or idiopathic condition are the various etiologies relating to Preiser disease. Radial-sided wrist pain without any history of trauma is the usual complaint. It is difficult to diagnose earlier on plain radiographs. Hence, MRI is done when the diagnosis is suspected. Although with a lack of definitive evidence, the underlying pathology is felt to be due to disruption of the blood supply of the scaphoid [2].

The scaphoid originates from the fusion of two chondrification zones, known as centralia, which fuse during the seventh week of intrauterine life when the crown-rump length reaches 50 mm [3]. The scaphoid lies at the radial border of the proximal carpal row and is elongated and boat-shaped. The blood supply to the scaphoid is mainly by the branches from the radial artery. The dorsal supply accounts for 70-80% of the internal vascularity of the scaphoid whereas the palmar supply accounts for the rest [4]. The stages of avascular necrosis of scaphoid are cellular death of bone components, secondary to ischemia, followed by the consequent collapse of bone components which results in pain and loss of function. The main aim of treatment is to relieve pain, regain the range of motion of the wrist joint, and delay the onset of arthritis.

## Case presentation

**Figure 1:**
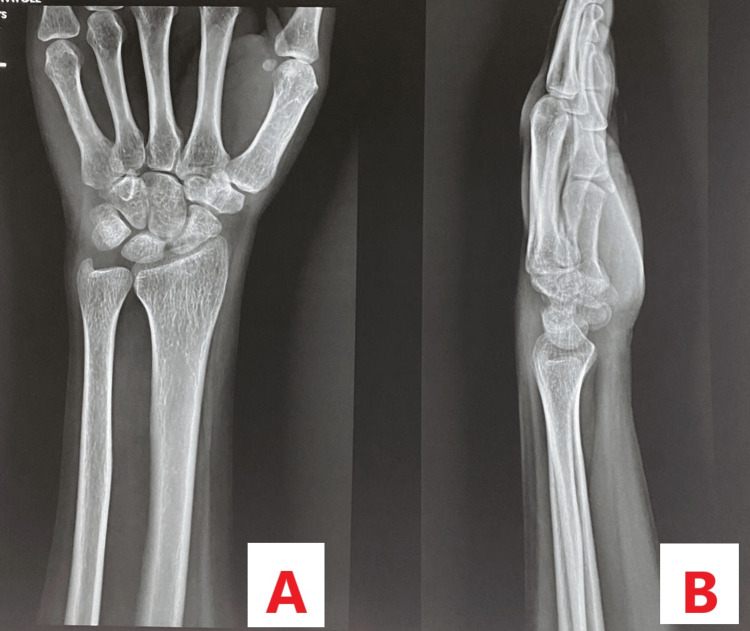
Plain radiograph of the left wrist joint showing no abnormality. A: Plain radiograph showing the posteroanterior view. B: Plain radiograph showing the lateral view.

A 23-year-old female patient came to our outpatient department with complaints of pain over the left wrist joint for seven months. The patient did not give any history of trauma. Clinically, the patient had tenderness over the anatomical snuff box, and the range of motion was normal but painful. The patient did not give any history of similar complaints in the past. A plain radiograph of the left wrist joint was done which was suggestive of no abnormality, as shown in Figure 1.

**Figure 2:**
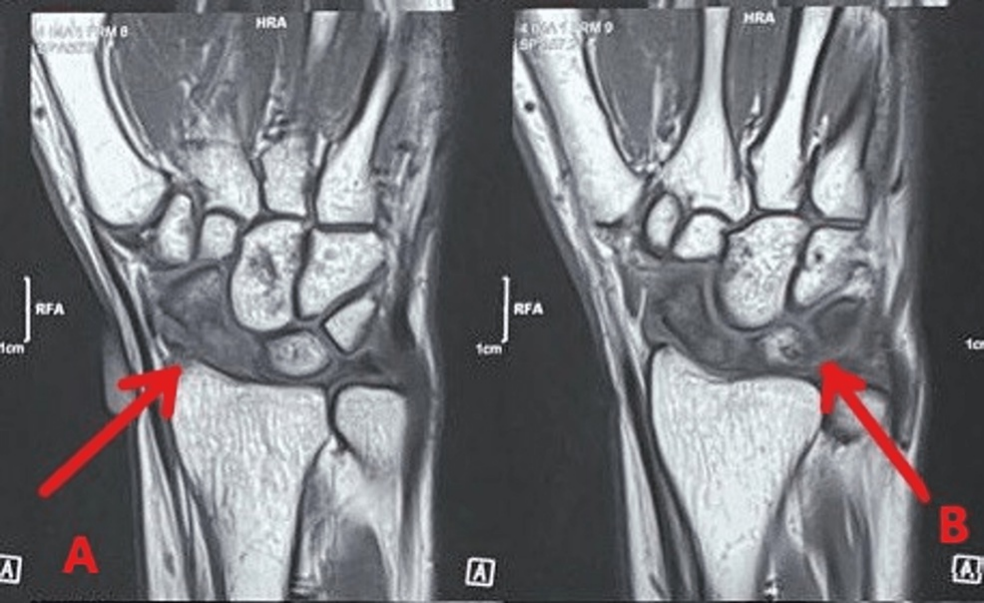
MRI showing diffuse altered marrow signal of the scaphoid. A: MRI showing altered signal in the scaphoid. B: MRI showing altered signal in lunate and triquetrum.

MRI was suggestive of diffuse altered marrow signal in the scaphoid suggestive of avascular necrosis along with altered marrow signals in lunate and triquetrum bones, as shown in Figure 2.

**Figure 3:**
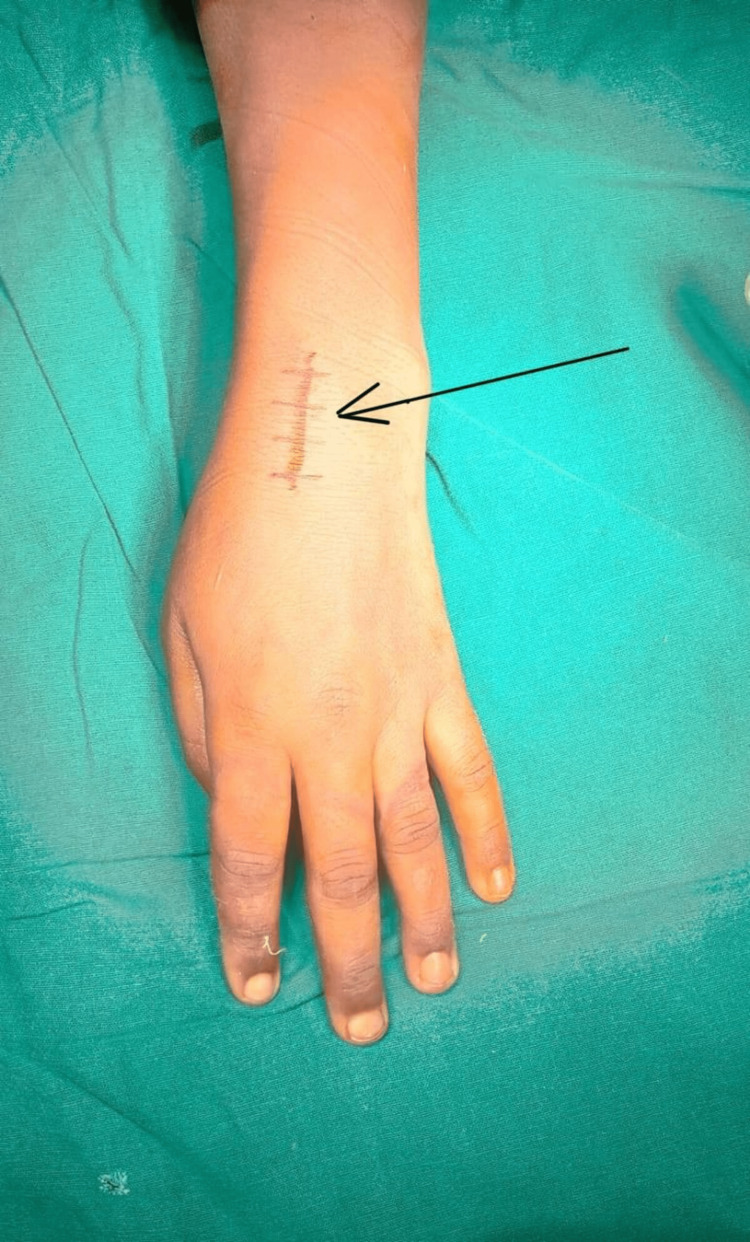
Incision marked over the dorsal aspect of the wrist.

The patient was planned for a proximal row carpectomy. Tourniquet was applied and the limb was exsanguinated after routine prepping and draping. A 3 cm incision was taken over the dorsal surface of the wrist joint over the second extensor compartment after palpating the scaphoid and radial styloid, as shown in Figure 3.

**Figure 4:**
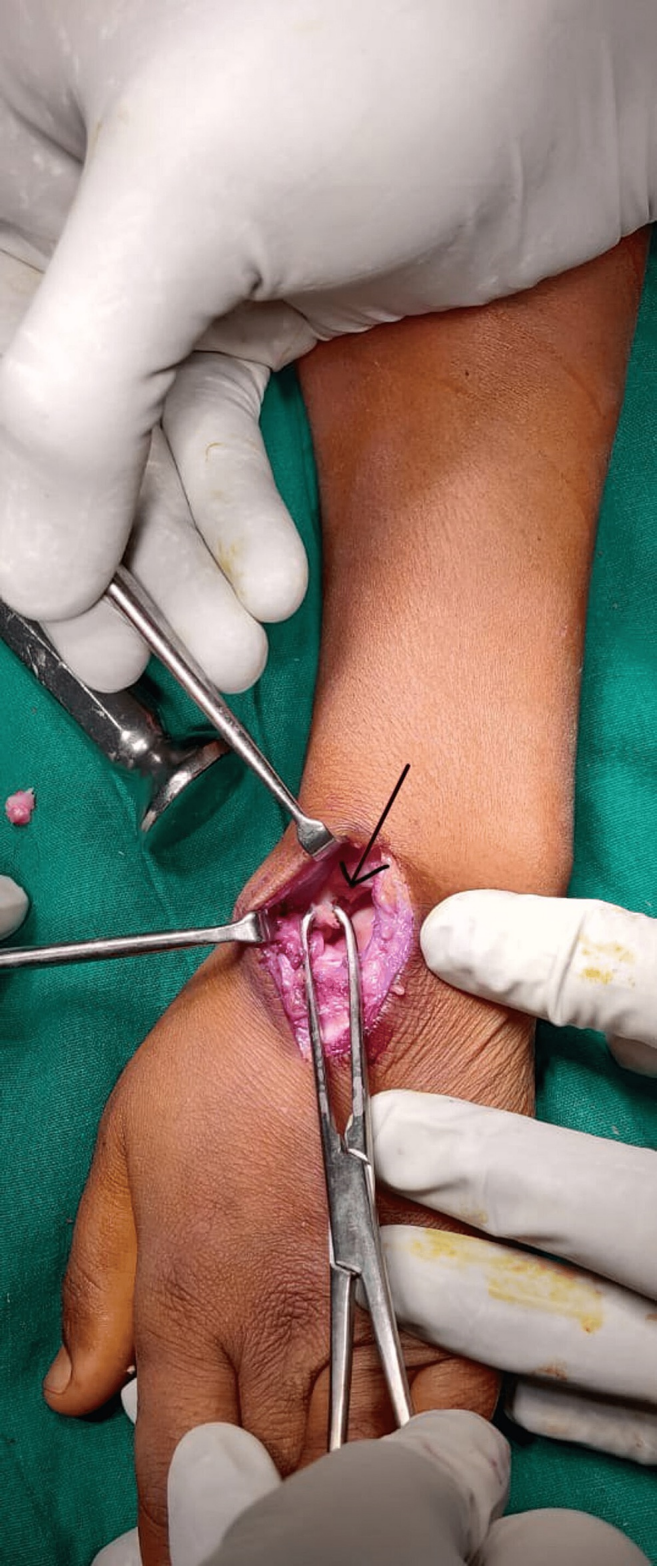
Intraoperative picture showing excision of the proximal carpal bones.

Scaphoid, lunate, and triquetrum were visualized and excised, creating a neo-articulation between the capitate and lunate fossa of the radius, as shown in Figure 4. Routine closure was performed, and the tourniquet was deflated after applying pressure dressing.

**Figure 5:**
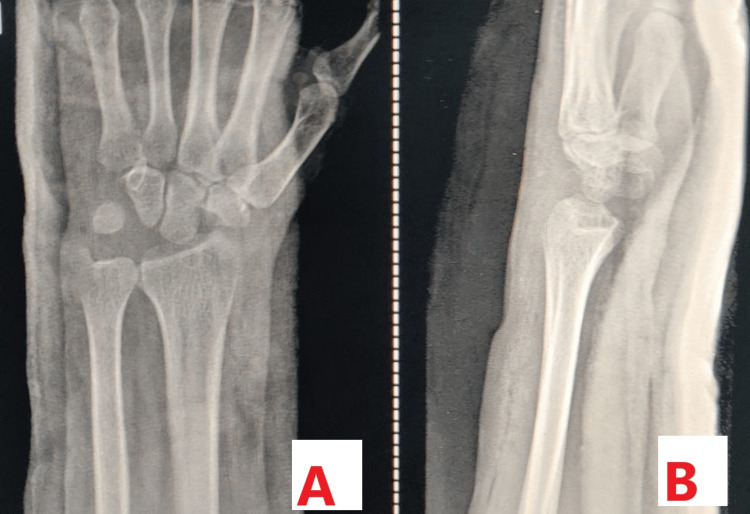
Postoperative radiograph of the wrist joint. A: Posteroanterior view of the wrist joint. B: Lateral view of the wrist joint.

A postoperative radiograph was taken, as shown in Figure 5.

A wrist splint was given for one week, and range of motion exercises were started after one week. The wound dressings were done on postoperative days two, eight, and 11, and sutures were removed on day 14. The wound went on to heal with primary intention. Full range of motion was regained three weeks postoperatively with no complaints of pain.

## Discussion

Our patient presented with a seven-month history of radial side wrist pain in the absence of any antecedent trauma. There was tenderness over the scaphoid, and the radiograph showed no evidence of any scaphoid fracture. This ruled out proximal pole avascular necrosis of the scaphoid which is usually secondary to a fracture. After establishing the diagnosis, the disease was radiologically classified as it is relevant to the expected outcome of the patient. Herbert and Lanzetta classified Preiser disease into four stages based on the area of scaphoid involved on the plain radiograph [5]. A majority of the literature suggests that this disease is due to diffuse involvement of the scaphoid. Herbert and Lanzetta proposed that the process begins in the proximal pole which then advances to the remaining scaphoid. Recently, another classification by Kalainov described two types of Preiser disease based on the degree of scaphoid involved in MRI [6]. Our patient had type 1 Preiser disease according to Herbert and Lanzetta classification and type 2 Preiser disease according to Kalainov classification.

Preiser disease is idiopathic avascular necrosis of the scaphoid bone. Treatment of Preiser disease is controversial with no prospective long-term trials comparing the different treatment options. Thus, treatment is largely a matter of surgeon preference. Nonoperative or conservative management includes immobilization, non-steroidal anti-inflammatory drugs, and electrical stimulation. Operative procedures can be scaphoid preserving or sacrificing surgeries. Scaphoid-preserving surgeries include closing wedge radial osteotomy [7], curettage with or without bone graft [8], vascularized bone grafting [9], arthroscopic drilling, and arthroscopic debridement [10]. Salvage procedures are indicated in only Herbert stage one or two where the cartilage is still preserved.

Proximal row carpectomy is a scaphoid-sacrificing procedure that helps to regain an almost complete range of motion of the wrist joint with no residual disability [11]. The proximal row carpectomy involves the removal of the intercalary proximal carpal row turning the wrist into a simple hinge joint. This procedure creates a new radio capitate articulation, unloads the areas of arthrosis, and is a movement-preserving treatment that can withstand compressive stresses over time [12]. Different indications for this procedure are Kienbock disease, degenerative arthrosis secondary to scapholunate advanced collapse deformity or chronic scaphoid non-union and chronic peri-lunate dislocations and fracture-dislocations [13]. It is a useful procedure for gaining a near-complete wrist range of motion. The articular surfaces of the lunate fossa of the radius and the capitate should be free of arthritic changes for this procedure to succeed. Our patient had complete relief from pain and regained near complete range of motion of the wrist joint. Thus, the stage of the disease and the timing of surgery play an important prognostic role in proximal row carpectomy.

## Conclusions

This case report showed the presence of avascular necrosis of the scaphoid without any antecedent trauma. This idiopathic avascular necrosis of the scaphoid is also known as Preiser disease. Treatment options for Preiser disease depend on the age of the patient, the duration of symptoms, and the presence of viable cartilage in the surrounding carpals.

Proximal row carpectomy has earned a respectable place in the arsenal of hand surgeons and is a good treatment option for Preiser disease. As a salvage procedure for wrist arthritis due to scaphoid avascular necrosis, it shows good results concerning patient satisfaction and regaining a near-complete range of motion of the wrist joint with minimal complications. However large-scale studies are required to determine the most efficient method for treating Preiser disease.
